# Effect of Frequency and Ratio of Wet/Dry Stages in Cyclic Corrosion Tests on Localized Corrosion of Complex-Phase High-Strength Steel

**DOI:** 10.3390/ma16237329

**Published:** 2023-11-24

**Authors:** Jin-Seok Yoo, Geon-Il Kim, Jung-Gu Kim

**Affiliations:** Department of Materials Science and Engineering, Sungkyunkwan University, 2066, Seobu-Ro, Jangan-Gu, Suwon-Si 16419, Republic of Korea; wlstjr5619@skku.edu (J.-S.Y.); geonil1027@skku.edu (G.-I.K.)

**Keywords:** advanced high-strength steel, complex-phase, atmospheric corrosion, cyclic corrosion test, iron oxide

## Abstract

This study delves into the atmospheric corrosion behavior of chromium-free complex-phase (CP) steel, specifically investigating the influence of wet/dry frequency and ratio in cyclic corrosion tests (CCT). The study employs a modified ISO 14993 standard CCT method, which involves salt spray, dry, and wet stages. After 15 and 30 CCT cycles, mass loss, maximum corrosion depth, and corrosion products were analyzed to gain insights into corrosion mechanisms. In general, increasing the frequency and wet/dry stage ratio in CCT extends the time for autocatalytic reactions to occur, leading to accelerated localized CP steel corrosion and increased pitting factors. However, as the rust layer thickens, uniform corrosion may also intensify, so careful considerations are necessary. This study underscores the importance of controlling the frequency and ratio of wet/dry stages in CCT for effectively analyzing localized corrosion behavior in specimens.

## 1. Introduction

Advanced high-strength steels (AHSS) have garnered significant attention in the automotive industry due to their remarkable strength-to-weight ratio. The use of AHSS in automotive applications promises to reduce fuel consumption, lower greenhouse gas emissions, and enhance passenger safety [1,2,3]. However, the relatively thinner thickness of AHSS compared to traditional automotive steel sheets, which contributes to its weight-saving advantages, presents a significant vulnerability in the form of increased corrosion susceptibility. In particular, complex-phase (CP) steel, a subtype of AHSS, is highly sensitive to corrosion. It is composed of various alloying elements and multiple phases with varying electrochemical potentials, such as ferrite, bainite, and martensite [4]. These variations can lead to micro-galvanic and localized corrosion issues in CP steel under atmospheric conditions. Notably, recent studies have reported that chromium (Cr) can increase localized corrosion in low-alloy steels under atmospheric corrosion conditions [5,6,7]. To address this concern, CP steel variants without Cr have been developed. However, there have been few evaluations of the atmospheric corrosion behavior of these Cr-free CP steel variants.

The cyclic corrosion test (CCT) stands as one of the most widely accepted and representative corrosion tests in atmospheric corrosion studies. Its popularity stems from its ability to provide insights into material interactions with corrosion products under realistic environmental conditions [8,9,10,11]. CCT conditions subject materials to various corrosion parameters, including salt concentration, time of a cycle, humidity, and temperature, thereby enabling the assessment of atmospheric corrosion behavior within specific environmental contexts. However, this test comes with a significant drawback of being time-consuming. Notably, the dry stage in CCT is essential, yet it can significantly prolong the test duration, as once the solution is completely dry, corrosion no longer proceeds. Thus, effectively managing the frequency and duration of the dry stage offers a promising approach to shorten CCT testing periods.

Therefore, this study aims to analyze the corrosion behavior of CP steel without Cr elements using electrochemical methods and modified cycle periods within CCTs. The electrochemical methods provide insights into electrochemical properties in salt spray stages, encompassing potentiodynamic polarization tests and electrochemical impedance spectroscopy (EIS) conducted on the CP steel specimen. Simultaneously, the CCTs explore the impact of corrosion product interactions on the corrosion behavior of CP steel in atmospheric conditions, incorporating variations in the frequency and ratio of wet/dry stages. Additionally, microscopic analysis, employing optical microscopy (OM), atomic force microscopy (AFM), scanning electron microscopy (SEM), X-ray diffraction analysis (XRD), and electron probe microanalysis (EPMA), explores the effects of different wet/dry stage frequencies and ratios within CCT.

## 2. Materials and Methods

### 2.1. Specimens, Solution, and Microstructure Analysis

The AHSS material used in this study was CP steel sheets with Cr-free as indicated in Table 1. This specimen was manufactured by POSCO in Pohang, South Korea, and exhibited an ultimate tensile strength of approximately 1200 MPa with a tensile elongation of about 15%. For the CCT tests, the specimens were machined into dimensions of 135 mm × 75 mm × 1.2 mm, and for investigating their electrochemical properties, 15 mm × 15 mm × 1.2 mm samples were prepared using wire cutting. Prior to testing, the specimen surfaces were meticulously polished using 600-grit silicon carbide paper and then rinsed with ethanol and distilled water. The test solution used in this study was a 5 wt.% NaCl solution (pH 6.14), following the ISO 14993 standard [12]. To examine the microstructure of the specimens, AFM (NX10, Park Systems, Suwon, Republic of Korea) was utilized to analyze the surface topography and potential of each phase of the specimen using scanning Kelvin probe force microscopy (SKPFM) mode using the AFM tip (MagneticMulti75E-G). OM (LEICA 300, Leica Microsystems, Langen, Germany) was employed after etching the specimens with a Nital solution (5 mL 60% HNO_3_ solution + 95 mL ethanol) and LePera solution (50 mL 1% Na_2_S_2_O_5_ solution in distilled water + 50 mL 4% dry picric acid in ethanol) to reveal each phase.

### 2.2. Electrochemical Tests

All electrochemical tests in this study were conducted at room temperature using a three-electrode setup within a 1000 mL Pyrex glass corrosion cell. The specimens were connected to the working electrode, with a saturated calomel electrode (SCE) serving as the reference electrode, while two glassy carbon bars were utilized as the counter electrode. To ensure consistent conditions, the area of specimen was maintained to 1 cm^2^ with silicone epoxy, and an open-circuit potential (OCP) was measured over an 8 h period before the electrochemical tests commended. The electrochemical tests were performed using a VSP- 300 instrument (Bio-Logic SAS, Seyssinet-Pariset, France). Potentiodynamic polarization tests were carried out at a scan rate of 0.166 mV/s, covering a scan range from −300 mV vs. OCP to 1600 mV_SCE_. EIS was conducted across a frequency range spanning from 100 kHz to 10 mHz, with an amplitude of ±10 mV vs. OCP. The impedance data were subsequently analyzed and fitted using ZSimpWin software (ZsimpWin 3.21, EChem Software, Ann Arbor, MI, USA).

### 2.3. Cyclic Corrosion Tests and Corrosion Product Analysis

**Figure 1:**
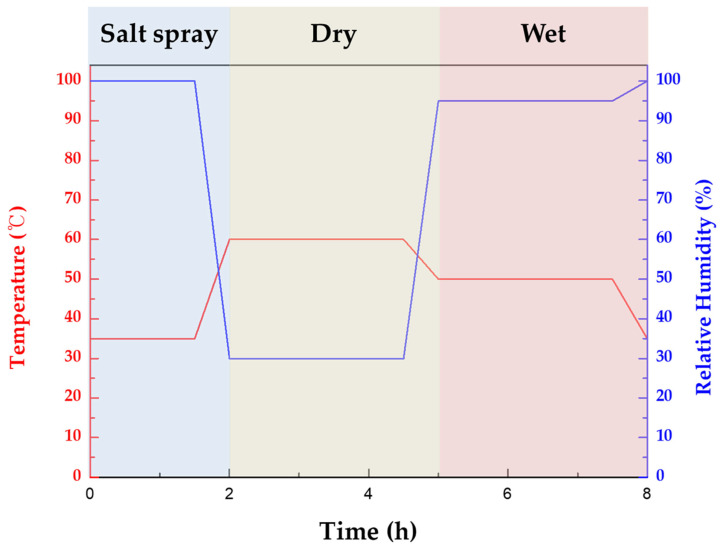
The schematic of a single cycle of the 1st condition CCT.

In this study, we conducted CCT experiments using CRH-1100 (Q-LAB, Cleveland, OH, USA) to analyze how the frequency and ratio of wet/dry stages in a single cycle affect corrosion behavior. The area of the CCT specimen was controlled to 70 mm × 130 mm (91 cm^2^) with silicone tape. Figure 1 illustrates the single-cycle procedure for the 1st condition of CCT, which was adapted from the ISO 14993 standard. Each complete CCT cycle of this condition had a duration of 8 h, consisting of a 2 h salt spray phase at 35 °C, followed by a 3 h dry stage at 60 °C with a relative humidity of 30%, and concluding with a 3 h wet stage at 50 °C with a relative humidity of 95%. The ramp time between each stage was set to 30 min. In the 2nd through to the 4th CCT condition, the wet/dry stage of the cycle was further divided into two, and the ratio of wet/dry stages were adjusted to 1, 2, and 3 with the same temperature, relative humidity, and ramp time, as detailed in Table 2. The CCT specimens were placed in the CCT chamber at an inclination of 70 degrees from the floor. To check the evaporation time of the water film in the dry stage, a cell was manufactured as shown in Figure S1a,b. We analyzed the mass loss and maximum corrosion depth of specimens subjected to 15 and 30 cycles of CCT under each condition, after removing the corrosion products through a process involving sandblasting and subsequent acid cleaning, using a 15 wt.% HCl and 0.35 wt.% hexamethylenediamine solution. After acid cleaning, the surface of the specimen was photographed, and localized corrosion density and area fraction were calculated using Image J software (Image J 1.49, National Institutes of Health, Bethesda, MD, USA). The mass values were measured using a precision scale (CAW-220, CAS, Yangju, Republic of Korea) with a linearity of 0.2 mg. The initial weight was measured before area control, and the final weight was measured after CCT with all corrosion products removed. The results of maximum corrosion depth were measured using a digital point micrometer (IP65 342-252-30, Mitutoyo, Kawasaki, Japan) with a resolution of 2 μm as depicted in Figure S2a. The maximum corrosion depth was determined by analyzing the residual thickness after the CCT testing of the specimen from which corrosion products were removed. The specimens were divided into 15 parts, and the deepest region of each part was measured, as shown in Figure S2b. Among them, the smallest value was subtracted from the initial thickness (1.2 mm) to calculate the maximum corrosion depth. All CCT tests were repeated once again, and the mass loss value was the average, while the maximum corrosion depth was the maximum value among two specimens.

Following the CCT analysis, the corrosion products were examined using XRD (SmartLab, Rigaku, Tokyo, Japan) and SEM with EPMA mode (JXA-8530F, JEOL Ltd., Akishima, Japan) after rinsing with distilled water and drying with nitrogen gas. XRD was conducted to identify corrosion products after CCTs, and SEM and EPMA were used to analyze the cross-section of the corrosion products to investigate the corrosion mechanisms between the corrosion products and the specimens.

## 3. Results and Discussion

### 3.1. Microstructure Analysis

**Figure 2:**
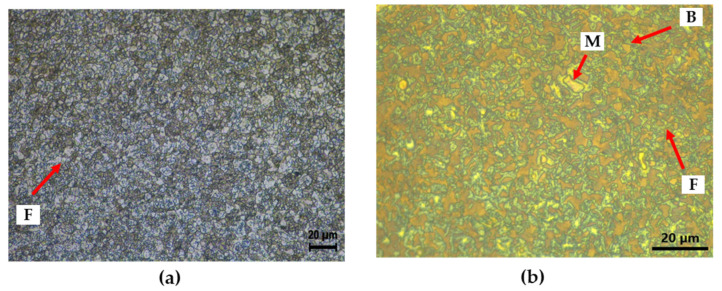
Microstructure image of CP steel with OM: (**a**) Nital etching and (**b**) LePera etching; F—ferrite, M—martensite, B—bainite.

In order to distinguish the phases of the specimen, microstructure analysis was performed through OM after etching with Nital and LePera solutions. Figure 2a presents OM image of the CP steel after Nital etching for 20 s. In the image, the white color representing the ferrite phase was clearly visible, but it was difficult to distinguish martensite and bainite [13]. To clarify the phases, LePera etching was performed to dye each phase with a more distinct color. As a result, it was confirmed that the microstructure of CP steel was clearly divided into blue ferrite, white martensite, and brown bainite, as shown in Figure 2b [14,15,16].

**Figure 3:**
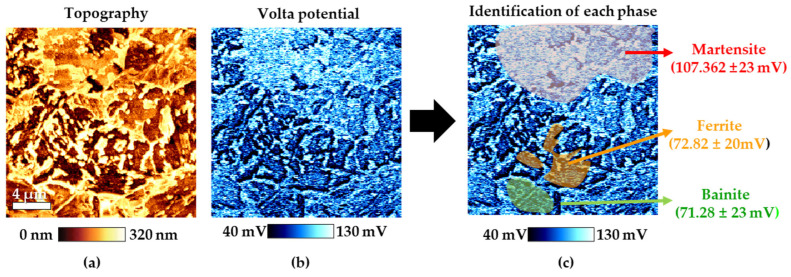
Results of AFM after Nital etching of CP steel: (**a**) topography, (**b**) Volta potential and (**c**) phase classification.

Figure 3a,b present the results of AFM analysis of CP steel specimen after Nital etching, depicting surface topography and potential using AFM, respectively. In the topography after Nital etching, areas with noticeable low height, representing the most etched ferrite in the Nital solution among the three different phases, were identified [17]. Additionally, based on previous research, martensite is known to have a higher Volta potential compared to ferrite, while ferrite and bainite have similar Volta potentials [18,19]. These results confirm that the red area corresponds to martensite, the yellow area to ferrite, and the green area to bainite. As shown in Figure 2 and Figure 3, the microstructure of CP steel has different phases with different potentials. Due to these characteristics, it has a negative effect on corrosion resistance because of micro-galvanic corrosion.

### 3.2. Electrochemical Tests

**Figure 4:**
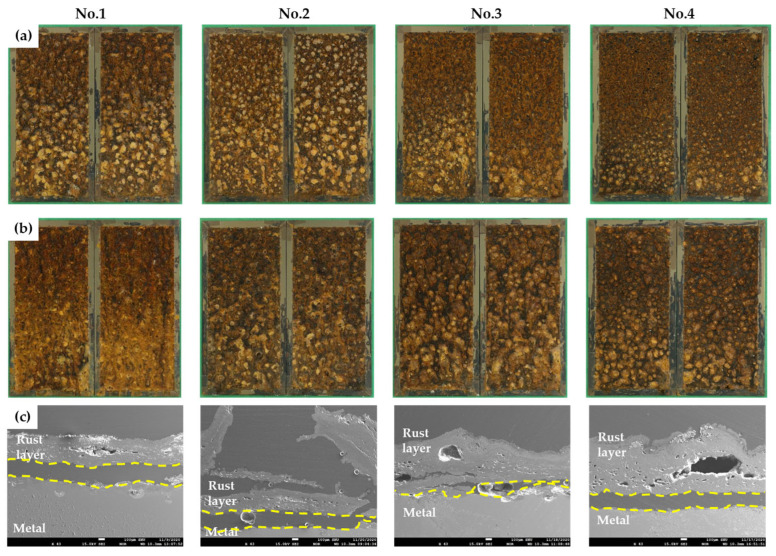
The rust layers after CCT for four different conditions: (**a**) surface images after 15 cycles, (**b**) surface images after 30 cycles, and (**c**) SEM images of cross-section after 30 cycles (yellow line: interface between corrosion product and metal).

Figure 4a,b are surface photographs after 15 and 30 cycles of CCT in each condition, and Figure 4c are SEM cross-sectional images after 30 cycles of CCT. As shown in the surface images, the rust layers are very rough and porous, and the cross-sectional images also show that there are many pores and cracks in the rust layers. Therefore, in the salt spray stage, the sprayed solution would have reached the steel surface, creating an immersion state. It is worth noting that the corrosion rate is approximately five times faster in the actual salt spray stage due to the smooth supply of oxygen [20]. Despite this, the corrosion behavior of the specimen in the salt spray stage was analyzed using electrochemical methods, considering the similarity in corrosion mechanisms to the immersion state.

**Figure 5:**
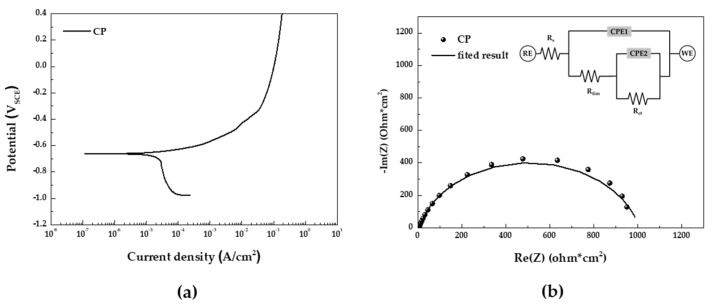
Results of electrochemical analyses in 5 wt.% NaCl solution: (**a**) potentiodynamic polarization curve and (**b**) EIS Nyquist plot and equivalent electrical circuit for fitting.

The potentiodynamic polarization test in the 5 wt.% NaCl solution of CP steel depicted in Figure 5a is summarized in Table 3. In the potentiodynamic polarization curve, we observed a low anodic polarization slope (β_a_) and a large cathodic polarization slope (β_c_), indicating a typical corrosion behavior controlled by the oxygen diffusion rate of carbon steel. Figure 5b display the impedance spectra in the form of Nyquist plots and the equivalent electrical circuit used for fitting the EIS data. The results of the EIS fitting parameters are summarized in Table 4. R_s_ represents the solution resistance, and the constant phase element (CPE) denotes a non-ideal capacitance. CPE1 corresponds to the oxide film capacitor, with Q_flim_ indicating its magnitude. R_film_ represents the electrical resistance of the oxide film. CPE2 symbolizes the capacitor formed by the electric double layer between the solution and the surface interface, and Q_dl_ represents the magnitude of CPE2. R_ct_ stands for the charge transfer resistance for metal dissolution. The parameter ‘*n*’ is an experimental exponent characterizing ideal capacitive behavior (0 ≤ *n* ≤ 1), and R_p_ represents the polarization resistance, which combines R_film_ and R_ct_. The impedance of the CPE is described by Equation (1). *Y*_0_ is the coefficient of proportionality with units of F·s^(n−1)^, *ω* is the angular frequency with units of rad·s^−1^, and *i*^2^ = −1 defines the imaginary number ‘*i*’ [21,22,23].

(1) $$Z_{CPE}=Y_{0}^{-1}{\left(j\omega \right)}^{-n}$$

As a result of EIS analysis, when comparing R_film_ and R_ct_ of CP steel, R_film_ has low resistance. This suggests that the oxide film is ineffective at suppressing corrosion, leading to active corrosion behavior. Therefore, by combining the potentiodynamic polarization and EIS results, it can be confirmed that uniform corrosion of the specimen takes place in the salt spray stage of CCT.

### 3.3. Effect of Frequency and Ratio of Wet/Dry Stage in the CCT

**Figure 6:**
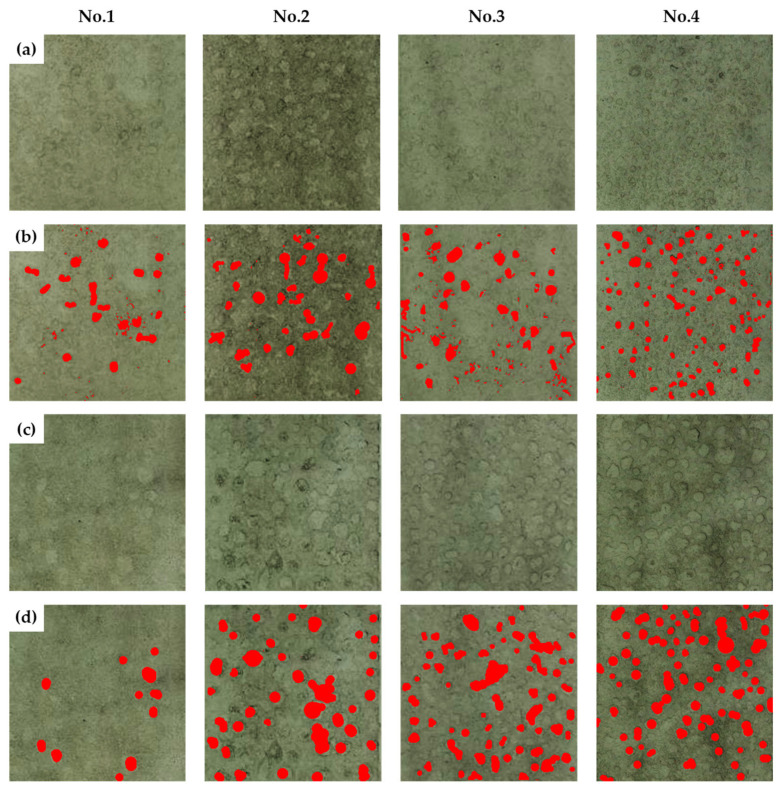
Results of CCT for four different conditions after acid cleaning (**a**) surface images after 15 cycles, (**b**) localized corrosion area after 15 cycles, (**c**) surface images after 30 cycles, and (**d**) localized corrosion area after 30 cycles.

All images after CCT and raw data related to mass loss and corrosion depth measured during the CCT have been incorporated into Figure S3 and Tables S1 and S2. Figure 6a,c illustrate the surface conditions of the specimens after 15 and 30 cycles of CCT with acid cleaning, while Figure 6b,c exhibit the localized corrosion area using Image J software after 15 and 30 cycles. The results of localized corrosion density and area fraction are summarized in Table 5. To calculate the corrosion density, the area of surface images was standardized to 70 mm × 70 mm (49 cm^2^), considering the number of pits and the area fraction where localized corrosion occurred. By comparing surface images, localized corrosion densities, and area fraction values for each CCT condition after 15 and 30 cycles, it becomes evident that as the frequency and ratio of wet/dry stages increase, localized corrosion becomes more pronounced.

**Figure 7:**
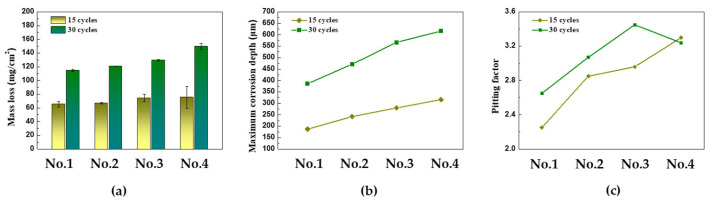
Results of CCT for four different conditions after acid cleaning: (**a**) mass loss, (**b**) maximum corrosion depth, and (**c**) pitting factor.

Figure 7a–c illustrate the mass loss, maximum corrosion depth, and pitting factor for each CCT condition. The effect of frequency and ratio of wet/dry stages can be clearly seen in the data shown in the graphs. The mass losses in 15 cycles were found to be 65.4, 66.8, 74.5, and 75.48 mg/cm^2^, and the maximum corrosion depth values were 187, 242, 280, and 316 μm from the 1st condition to the 4th condition, respectively. To provide a better perspective, the mass loss values can be converted into a uniform corrosion thickness using Equation (2).

(2) $$Uniform\;corrosion\;depth\;(\mu m)=\frac{Mass\;loss\;\left(\frac{mg}{cm^{2}}\right)}{Densisy\;of\;Iron\;\left(\frac{mg}{cm^{3}}\right)}*\;10,000$$

Considering that the density of iron at room temperature is 7874 mg/cm^3^, the uniform corrosion depths under each condition are calculated as 83.4, 84.8, 94.6, and 95.86 μm, respectively. Additionally, the pitting factors were calculated using Equation (3).

(3) $$pitting\;factor=\frac{Maximum\;corrosion\;depth}{Uniform\;corrosion\;depth}$$

The pitting factors of each condition were calculated as 2.25, 2.85, 2.96, and 3.30, respectively. These calculations confirm that as the frequency and ratio of wet/dry stages increased in the CCT up to 15 cycles, the localized corrosion increased. In particular, the occurrence of localized corrosion increased more significantly with an increase in the frequency of the wet/dry stages.

In this study, CCT from 15 to 30 cycles is referred to as the ‘later stages’. The mass losses in 30 cycles were found to be 114.6, 120.7, 129.3, and 149.7 mg/cm^2^, and the maximum corrosion depth values were 386, 471, 567, and 616 μm from the 1st condition to the 4th condition, respectively. These results can be converted to a uniform corrosion depth and pitting factor through Equations (2) and (3). The uniform corrosion depth and pitting factor of each condition after 30 cycles were calculated as 145.5, 153.3, 164.2, and 190.1 μm and, 2.65, 3.07, 3.45, and 3.24, respectively. What is noteworthy is that the pitting factor in the 4th condition decreased compared to the 3rd condition in 30 cycles. This suggests that the electrolyte film created in the salt spray and/or wet stages on the surface did not completely evaporate in the dry stages due to the thicker rust layer. Therefore, after 30 cycles in the 4th condition, the overall corrosion of the specimen became more active, resulting in a decrease in the pitting factor.

**Figure 8:**
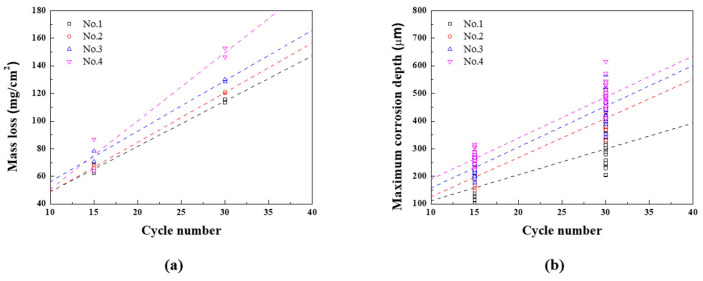
Results of CCT and regression line (**a**) mass loss and (**b**) maximum corrosion depth of CP steel.

The mass loss and corrosion depth for each CCT condition are presented as regression equations, as demonstrated in Figure 8a,b, and summarized in Table 6. At this time, the slope of the mass loss regression equation for nth condition is called *A_n_*, and the slope of the corrosion depth regression equation for nth condition is called *B_n_*. The uniform corrosion acceleration coefficient (α_1_) and localized corrosion acceleration coefficient (α_2_) are calculated using Equations (4) and (5).

(4) $$\alpha_{1}=\frac{A_{n}-A_{1}}{A_{1}}*100$$

(5) $$\alpha_{2}=\frac{B_{n}-B_{1}}{B_{1}}*100$$

These are important parameters that quantify the changes in corrosion rates under various conditions. When the wet/dry frequency increased from once to twice while keeping the ratio fixed (1st and 2nd), both uniform and localized corrosion coefficients increased. α_1_ increased by approximately 9.7%, with α_2_ increasing by approximately 57.1%. These results suggest that increasing the frequency of wet/dry stages accelerates uniform corrosion and even more sensitive localized corrosion. When the ratio of wet/dry stages was extended to 2 and 3 with the same two frequencies (3rd and 4th), the impact on uniform corrosion coefficient became more significant, the local corrosion coefficient did not change significantly. α_1_ increased to 11.5% and 50.9%, while *α*_2_ increased to 63.8% and 64.7%. These findings highlight that extending the ratio of wet/dry stage accelerates the uniform and localized corrosion rate, but when the ratio is 3, the uniform corrosion is more accelerated.

### 3.4. Analysis of Corrosion Products and Cross-Section after CCT

**Figure 9:**
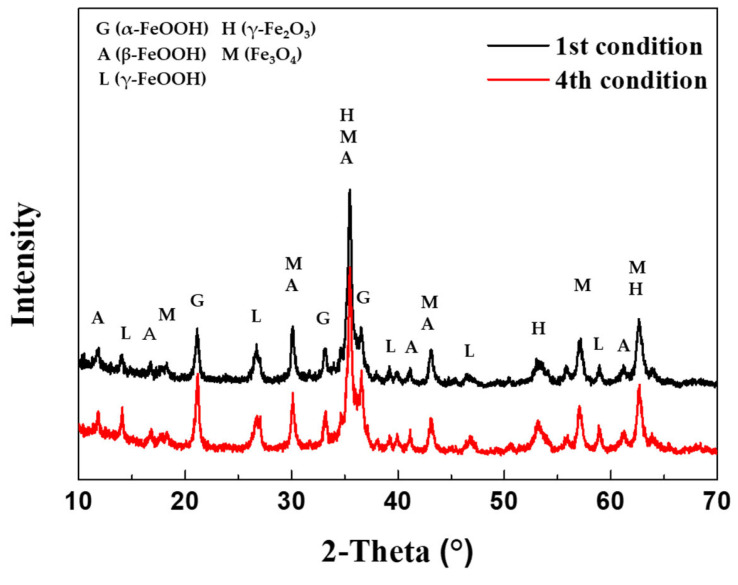
XRD analysis of corrosion products after 30 cycles of the 1st and 4th CCT conditions.

**Figure 10:**
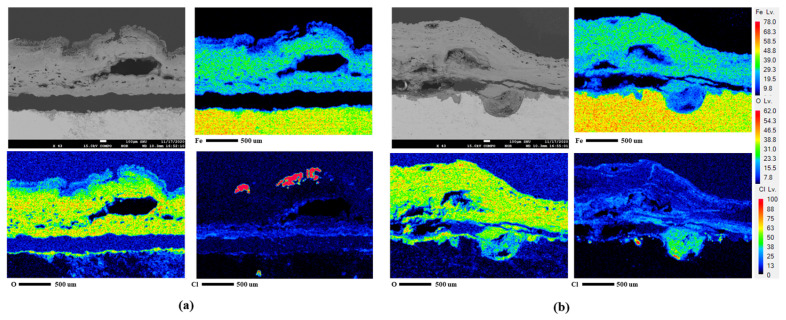
EPMA analysis after CCT for 30 cycles of CP steel: (**a**) 1st condition and (**b**) 4th condition.

The composition of the corrosion products generated after 30 cycles of the 1st CCT and 4th condition were analyzed using XRD and EPMA, as shown in Figure 9 and Figure 10a,b. As depicted in Figure 9, the corrosion products resulting from different CCT conditions were nearly identical. These products consisted of various hydroxides and oxides, including α-FeOOH (goethite), β-FeOOH (akaganeite), γ-FeOOH (lepidocrocite), γ-Fe_2_O_3_ (maghemite), and Fe_3_O_4_ (magnetite) [24,25,26]. The difference between the two conditions was revealed through a cross-sectional analysis with EPMA, as presented in Figure 10a,b. The EPMA images of the 4th condition, unlike the 1st condition, clearly show the localized corrosion area on the specimen surface and the high concentration of chlorine (Cl) there. The signal of Cl comes from akaganeite, a corrosion product that causes localized corrosion in wet/dry stages [27]. The details of localized corrosion mechanisms are discussed in the following section.

### 3.5. Mechanism of Localized Corrosion in CCT

#### 3.5.1. Corrosion Mechanism of Salt Spray and Dry Stages in the CCT 

**Figure 11:**
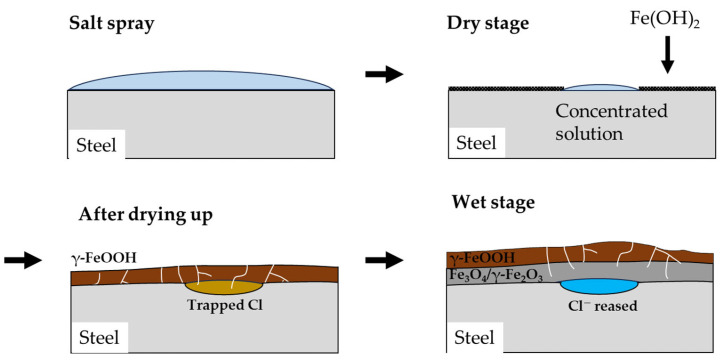
Schematic diagram of the mechanism of localized corrosion of CP steel under CCT.

The mechanism of corrosion in CP steel under CCT conditions is illustrated in Figure 11. The salt spray stage is similar to the immersion state as previously explained. The anode reaction of the corrosion is described by Equation (6), while the cathode reaction is represented by Equation (7) [28]. Then, the iron ion (Fe^2+^) diffuses into the water film and reacts with hydroxide ion (OH^-^) to produce Fe(OH)_2_, which is an unstable corrosion product, so it gradually dissolves in the water film, transforming into ferric oxyhydroxide, as shown in Equations (8) and (9) [29,30].

(6) $$Fe\to Fe^{2+}+{2e}^{-}$$

(7) 12O2+H2O+e−→2OH−

(8) $$Fe^{2+}+{2OH}^{-}\to Fe(OH{)}_{2}$$

(9) $$Fe(OH{)}_{2}\to FeOOH+O_{2}$$

During the dry stage, the salt spray solution evaporates and becomes concentrated in a specific area, and the corrosion rate continues to increase. Simultaneously, Fe(OH)_2_ coverts into porous goethite and lepidocrocite, while in the Cl-concentrated region, it transforms into akaganeite, a Cl^-^ trapped corrosion product [31,32,33]. What is noteworthy about the dry stage is that when the solution is completely dried, no corrosion reaction occurs. 

#### 3.5.2. Corrosion Mechanism of Wet Stage in the CCT 

In the subsequent wet stage, a water film forms on the surface, and trapped Cl in the akaganeite is released into the water film. At the same time, some of the lepidocrocite transforms into magnetite according to Equation (10), and these corrosion products act as cathodic sites due to their high conductivity [34]. Additionally, Oh et al. [35] reported that the magnetic maghemite also occurs as a corrosion product. Furthermore, in a confined space where Cl^−^ is released, hydrolysis of FeCl_2_ occurs, resulting in an autocatalytic reaction as depicted in Equation (11) [36,37]. Due to the increase in cathodic area and the decrease in pH, the corrosion of the specimen in the Cl-trapped region is accelerated, causing a localized corrosion of CP steel in the wet stage. This effect continues until the solution dries up in the dry stage or the water film is diluted on the surface by salt spray.

(10) $$8\gamma -FeOOH+{Fe}^{2+}+2e^{-}\to 3Fe_{3}O_{4}+4H_{2}O$$

(11) $${Fe}^{2+}+2H_{2}O+2Cl^{-}\to Fe(OH{)}_{2}+2HCl$$

#### 3.5.3. Effect of Frequency and Ratio of Wet/Dry Stage in the CCT

**Figure 12:**
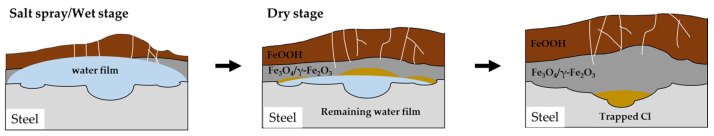
Schematic diagram of corrosion behavior of the 4th CCT condition in the later stages.

The impact of the frequency of wet/dry stages is quite evident in reducing wasted time during the dry stages. As depicted in Figure S1e,f, only 500 s are needed for the water film generated during the wet stage to fully evaporate. Any remaining time in the dry stage is essentially unutilized, as corrosion does not take place. Naturally, with an increase in CCT cycles, the duration required for the water film to entirely evaporate extends, influenced by the growing rust layer [38]. When the water film dries twice in one cycle, it effectively extends the duration for which the water film remains on the surface. Consequently, the time for the wet stage, as mentioned in the previous section, becomes longer, resulting in a deeper localized corrosion. For the same reason, the pitting factors also increase as the ratio of the wet/dry stages continues to rise, up to the 15th cycle. However, in the later stages, when the wet/dry ratio is set to 3 (4th CCT condition), uniform corrosion becomes more pronounced, leading to a decrease in the pitting factor. This suggests that the water film could not fully evaporate during the dry stages because the thickness of the corrosion product increased and the drying time was insufficient, as shown in Figure 12.

## 4. Conclusions

In this study, we analyzed the microstructure, electrochemical corrosion characteristics, and atmospheric corrosion behavior of Cr-free CP steel, a type of AHSS, with a varying frequencies and ratios of wet/dry stages. The investigation led to the following conclusions:

- The microstructure of CP steel consists of ferrite, bainite, and martensite with different surface potentials.
- The electrochemical measurements demonstrated that uniform corrosion occurred during the salt spray stage of CCT.
- With increasing frequency and ratio of wet/dry stages up to 15 CCT cycles, localized corrosion was accelerated more than uniform corrosion, resulting in an increase in the pitting factor.
- However, starting from the 15th CCT cycle, when the ratio of wet/dry stages was set to 3, uniform corrosion accelerated more than localized corrosion, leading to a decrease in the pitting factor because of the remaining water film.
- After conducting CCT for up to 30 cycles, we found that simulating localized corrosion was most effective when the frequency and ratio of wet/dry stages were both set to 2.

In conclusion, controlling the frequency and ratio of wet/dry stages during CCT is an effective method of accelerating localized corrosion in atmospheric conditions. However, it is important to select an appropriate dry stage duration as the number of CCT cycles increases. This study has provided insights into accelerating localized atmospheric corrosion by adjusting the frequency and ratio of wet/dry stages according to CCT conditions. As a result, we have confirmed a significant reduction in CCT time.

## Figures and Tables

**Table 1:** Concentration of main elements in the specimens (wt.%).

Specimens	Composition (wt.%)								
	C	Cr	Si	Mn	Ni	Al	Nb	Ti	Fe
CP steel	0.11–0.18	-	0.4–1.2	1.8–2.6	0.8–1.2	0.01–0.04	0.01–0.05	0.01–0.03	Bal.

**Table 2:** CCT conditions of a single cycle conducted in this study.

No.	Cycle Flow																																	
h.	1				2				3				4				5				6				7				8				Freq. (/Cycle)	Ratio (/Cycle)
min.	15	30	45	60	15	30	45	60	15	30	45	60	15	30	45	60	15	30	45	60	15	30	45	60	15	30	45	60	15	30	45	60		
**1**	Salt spray								Dry												Wet												1	1
**2**	Salt spray								Dry						Wet						Dry						Wet						2	1
**3**	Salt spray								Dry				Wet								Dry				Wet								2	2
**4**	Salt spray								Dry			Wet									Dry			Wet									2	3

**Table 3:** Potentiodynamic polarization test results in 5 wt.% NaCl solution.

Specimen	E_corr_ (V_SCE_)	i_corr_ (A/cm^2^)	β_a_ (mV_SCE_/de.)	β_c_ (mV_SCE_/de.)
CP steel	−0.660	2.29 × 10^−5^	47.8	859.1

**Table 4:** Parameters from EIS measurements.

Specimen	R_s_ (Ω × cm^2^)	CPE 1		R_film_ (Ω × cm^2^)	CPE 2		R_ct_ (Ω × cm^2^)	R_p_ (Ω × cm^2^)
		Q_film_ (Fs^n−1^/cm^2^)	n_1_		Q_dl_ (Fs^n−1^/cm^2^)	n_2_		
CP steel	4.06	3.20 × 10^−4^	0.86	10.23	2.31 × 10^−4^	0.85	994	1004

**Table 5:** Localized corrosion density and area fraction after CCT.

CCT Condition	No. 1		No. 2		No. 3		No. 4	
CCT Cycle	15 Cycles	30 Cycles	15 Cycles	30 Cycles	15 Cycles	30 Cycles	15 Cycles	30 Cycles
Localized corrosion density (#/49 cm^2^)	28	11	33	39	59	62	109	74
Localized corrosion area fraction (%)	4.87	3.23	8.63	13.97	5.24	15.69	9.36	17.21

**Table 6:** Regression coefficient of the corrosion data after CCT.

Specimen	CCT Condition	Uniform Corrosion [y (mg/cm^2^), x (Cycle Number)]	Localized Corrosion [y (μm), x (Cycle Number)]	α_1_ (%)	α_2_ (%)
CP steel	No. 1	y = 16.16 + 3.280 x, R^2^ = 99.19%	y = 18.67 + 9.036 x, R^2^ = 100.0%	-	-
	No. 2	y = 12.79 + 3.597 x, R^2^ = 99.96%	y = −15.77 + 14.20 x, R^2^ = 94.32%	9.7	57.1
	No. 3	y = 19.63 + 3.656 x, R^2^ = 98.94%	y = 9.90+ 14.80 x, R^2^ = 88.12%	11.5	63.8
	No. 4	y = 1.27 + 4.95 x, R^2^ = 95.12%	y = 40.90 + 14.88 x, R^2^ = 88.57%	50.9	64.7

## Data Availability

Data are contained within the article and supplementary materials.

## Supplementary Materials

The following supporting information can be downloaded at: https://www.mdpi.com/article/10.3390/ma16237329/s1, Figure S1: Cell information to check the evaporation time of the water film in the dry stage (a) photograph of the specimen, (b) schematic of the cell in the CCT chamber, (c) galvanic current during the 1 Cycle of CCT, (d) galvanic current during the first dry stage (salt spray to dry), (e) galvanic current during the second dry stage (dry to wet), (f) galvanic current during the third dry stage.; Figure S2: (a) Schematic of the digital point micrometer (IP65 342-252-30, Mitutoyo, Kawasaki, Japan), (b) divided regions to calculate the maximum corrosion depth.; Figure S3: Results of CCT for four different conditions after acid cleaning (a) 15 cycles, (b) 30 cycles.; Table S1: Raw data of mass loss after CCT.; Table S2: Raw data of corrosion depth after CCT (μm).
