# Supplementary material for: Effect of Frequency and Ratio of Wet/Dry Stages in Cyclic Corrosion Tests on Localized Corrosion of Complex-Phase High-Strength Steel
Source: Materials (Basel). 2023 Nov 24;16(23):7329. doi: 10.3390/ma16237329 (PMC10707363; doi:10.3390/ma16237329)
Supplement: Supplementary file 1 [file materials-16-07329-s001.zip › materials-2724975-supplementary.pdf]

materials

Supplementary materials

Effect of Frequency and Ratio of Wet/Dry Stages in Cyclic Corrosion Tests on Localized Corrosion of Complex-Phase High-Strength Steel

Jin-Seok Yoo, Geon-Il Kim and Jung-Gu Kim *

Department of Materials Science and Engineering, Sungkyunkwan University, 2066, Seobu-Ro, Jangan-Gu, Suwon-Si 16419, Republic of Korea; wlstjr5619@skku.edu (J.-S.Y.); geonil1027@skku.edu (G.-I.K.)
* Correspondence: kimjg@skku.edu

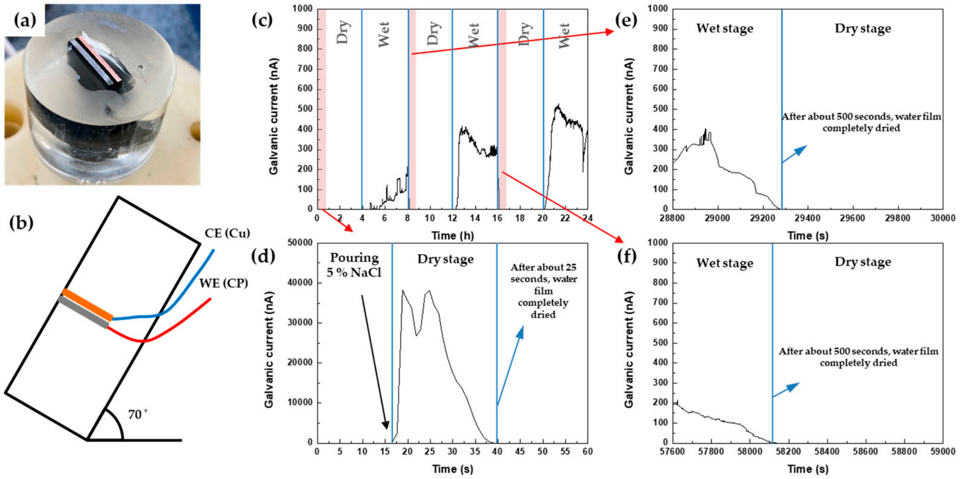

Figure S1. Cell information to check the evaporation time of the water film in the dry stage (a) photograph of the specimen, (b) schematic of the cell in the CCT chamber, (c) galvanic current during the 1 Cycle of CCT, (d) galvanic current during the first dry stage (salt spray to dry), (e) galvanic current during the second dry stage (dry to wet), (f) galvanic current during the third dry stage.

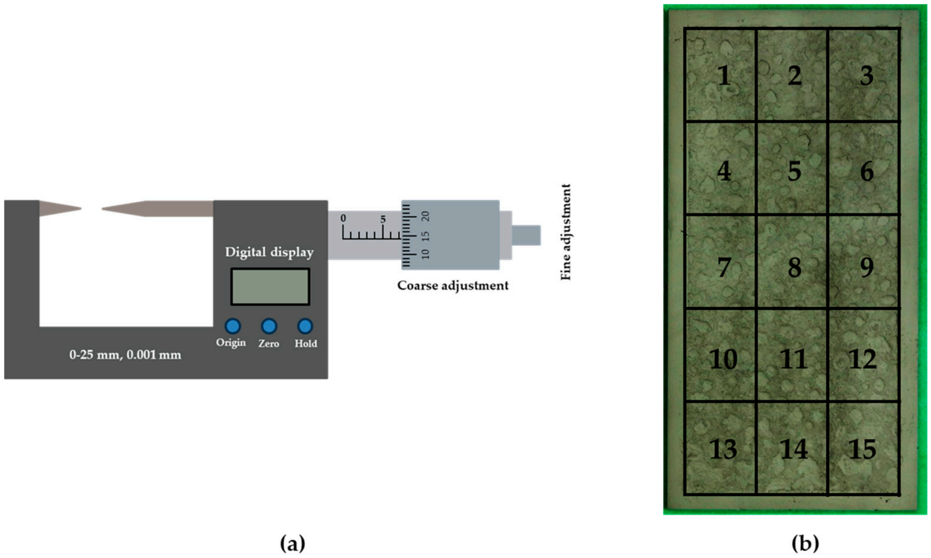

Figure S2. (a) Schematic of the digital point micrometer (IP65 342-252-30, Mitutoyo, Kawasaki, Japan), (b) divided regions to calculate the maximum corrosion depth.

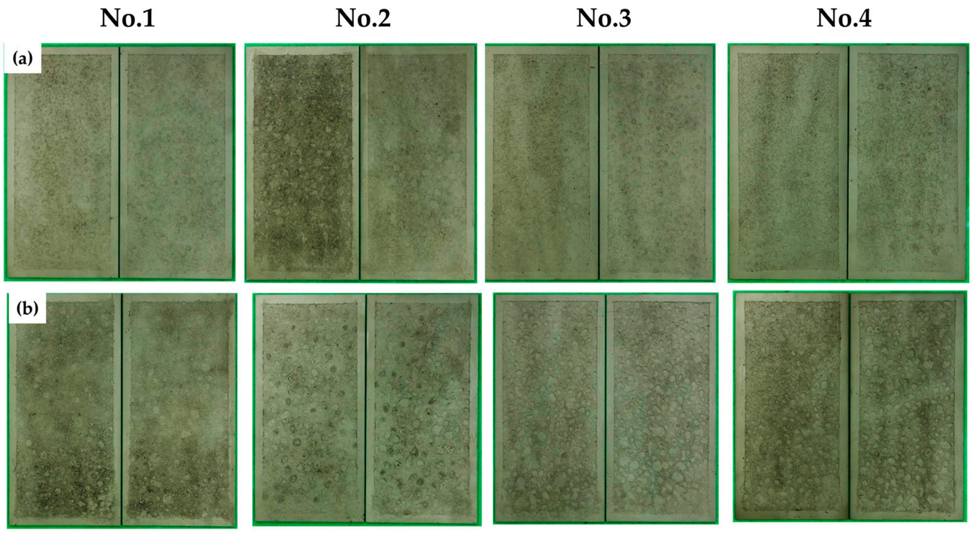

Figure S3. Results of CCT for four different conditions after acid cleaning (a) 15 cycles, (b) 30 cycles.

Table S1. Raw data of mass loss after CCT.

**CCT condition**	**No. 1**				**No. 2**				**No. 3**				**No. 4**			
**CCT cycle**	**15 cycles**		**30 cycles**		**15 cycles**		**30 cycles**		**15 cycles**		**30 cycles**		**15 cycles**		**30 cycles**	
**Initial mass (g)**	106.20	105.19	105.37	104.97	104.07	106.41	105.48	106.12	106.17	106.23	103.88	106.25	105.68	104.46	106.00	105.66
**Final Mass (g)**	100.77	99.21	95.05	94.44	97.93	100.40	94.53	95.10	99.75	99.10	92.19	94.40	97.78	98.64	92.08	92.33
**Mass loss (mg/cm^2)**	62.45	68.26	113.34	115.76	67.42	66.08	120.31	121.10	70.55	78.39	128.47	130.15	86.91	64.04	152.93	146.44
**Ave.**	65.36		114.55		66.75		120.71		74.47		129.31		75.48		149.69	
**Std.**	4.10		1.72		0.94		0.56		5.54		1.19		16.17		4.59	

Table S2. Raw data of corrosion depth after CCT (μm).

**CCT condition**	No. 1				No. 2				No. 3				No. 4			
**CCT cycle**	15 cycles		30 cycles		15 cycles		30 cycles		15 cycles		30 cycles		15 cycles		30 cycles	
**1**	187	161	318	258	200	184	436	468	255	279	567	477	272	256	545	545
**2**	162	177	289	258	236	211	430	433	272	228	523	470	282	278	499	544
**3**	161	165	243	282	176	189	403	471	214	279	457	515	264	256	479	616
**4**	171	169	204	282	203	205	394	381	254	249	467	489	248	254	491	490
**5**	147	174	253	234	216	190	392	380	268	234	416	400	254	227	506	465
**6**	172	148	375	229	242	199	409	436	228	233	538	454	248	228	512	532
**7**	174	183	386	286	216	200	368	412	221	234	469	439	244	270	499	572
**8**	178	150	311	206	203	211	404	441	211	226	416	448	271	253	529	453
**9**	169	148	313	278	210	182	407	413	244	229	507	496	282	253	501	541
**10**	149	146	298	330	185	196	422	402	225	225	492	408	304	316	492	485
**11**	154	136	293	366	214	200	424	408	232	280	422	419	268	267	451	467
**12**	172	154	303	338	223	164	405	443	202	231	479	492	271	257	413	489
**13**	114	095	295	302	202	156	410	397	176	199	437	342	289	246	410	411
**14**	141	139	361	258	191	149	341	433	203	219	420	352	287	224	413	443
**15**	128	102	296	247	198	167	330	416	192	214	417	387	309	244	413	411
**Ave.**	159	150	302	277	208	187	398	422	226	237	468	439	273	255	477	497
**Std.**	019	024	048	043	018	020	030	028	028	024	049	053	019	022	045	059
**Max.**	187	183	386	366	242	211	436	471	272	280	567	515	309	316	545	616
